# Diversity of genome size, endopolyploidy and SCoT markers in 20 *Trigonella* (Fabaceae) species

**DOI:** 10.1007/s13353-024-00886-9

**Published:** 2024-06-26

**Authors:** Monika Rewers, Agnieszka Lojko, Dorota Olszewska, Aleksandra Niklas, Iwona Jedrzejczyk

**Affiliations:** Department of Biotechnology, Bydgoszcz University of Science and Technology, Kaliskiego Ave. 7, 85-796 Bydgoszcz, Poland

**Keywords:** DNA content, Endoreduplication, Fenugreek, Flow cytometry, Molecular markers, Polysomaty

## Abstract

The *Trigonella* species possess medicinal, nutraceutical and pharmaceutical properties due to the presence of many bioactive compounds. Its therapeutic effects are mostly valuable in medicine, cosmetics and the functional food industry. Correct genetic characterisation of plant material is needed to increase the potential of *Trigonella* species by breeding and conservation programs. The aim of this study was to develop a reliable marker system to support the morphological and phytochemical analysis in *Trigonella* taxonomic research, species identification and characterization as well as determination of the interspecific variation within this genus along with relationships between species. For this purpose, flow cytometry and SCoT molecular markers were combined. Flow cytometric analyses revealed that *Trigonella* species possess very small and small genomes. The range of genome sizes was from 1.10 to 5.76 pg/2C, with most species possessing very small genomes (< 2.8 pg/2C). In seeds of 14 species endopolyploid nuclei were detected. Flow cytometric analysis of genome size enabled quick identification of four out of 20 species, while combined with endopolyploidy detection in seeds, facilitated distinction of the next seven species. ScoT molecular markers helped to identify closely related species with similar genome size and cell cycle activity. Therefore, flow cytometry was proposed as the first-choice method for quick accession screening, while the more detailed genetic classification was obtained using SCoT molecular markers.

## Introduction

The genus *Trigonella* L. belongs to the Fabaceae family. It consists of many perennial and annual species distributed in the dry regions around the Mediterranean Sea, West Asia, Europe, Africa, North America, and South Australia (Mabberley 1997). The exact number of species that comprise the genus *Trigonella* is still being analyzed, but the most relevant classification indicates 62 species in the genus (Dangi et al. 2016).

The *Trigonella* species have medicinal, nutraceutical and pharmaceutical properties due to the presence of many bioactive compounds (e.g. sapogenins, flavonoids, polysaccharide galactomannans, phenolics; Dangi et al. 2004; Ahari et al. 2009; Mehrafarin et al. 2011; Al-Asadi 2014). Within this genus, *T. foenum-graecum* (fenugreek) is the best-known species with the greatest economic importance since its long history in natural medicine and as a food condiment. High protein content and favourable amino acid composition make fenugreek seeds equal in nutritive value to soybean (Niknam et al. 2004). *Trigonella* seeds, particularly those of *Trigonella foenum-graecum* (fenugreek), have a variety of uses as food. Fenugreek seeds are widely used as a spice in culinary preparations. They add a distinct flavour to dishes and are commonly used in Indian, Middle Eastern, and Mediterranean cuisines. The seeds can be used as a food additive and preservative due to their flavour and potential antimicrobial properties. Fenugreek leaves and seeds can be used directly as a vegetable in various dishes (Syed et al. 2020; Ahmad et al. 2023). Moreover, it was proved that this plant has multiple therapeutic properties, mainly against atherosclerosis, hypertension, obesity, and cholestasis. The therapeutic potential in anti-cancerous, anti-inflammatory, antioxidant, antiulcer and antipyretic properties was also proved (Sushma and Davasena 2010; Naidu et al. 2011; Moradikor and Moradi 2013).

Taxonomic classification of species within the *Trigonella* genus was initially based on morphological characteristics. Sirjaev (1928–1932) divided the genus into three subgenera and 15 sections. Subgenus I: *Trigonella* sections: *Falcatulae, Callicerates, Uncinatae, Cylindricae, Samaroideae, Pectinatae, Erosae, Verae, Spinosae*. Subgenus II: *Trifoliastrum*: section *Capitatae*. Subgenus III: *Foenum-graecum* sections: *Foenum-graecum, Medicagoids, Medicago, Melilotus, Trifolium*. Nevertheless, karyogenetic and molecular data suggested that this taxonomic concept is insufficient because of the high variation of morphological features under environmental factors (Dangi et al. 2016; Al-Maamari et al. 2020). Therefore, environmentally independent karyological and molecular markers are searched to supplement morphological characterization. Among the karyological markers chromosome number and morphology, genome size and endopolyploidy level were used to support the taxonomic classification of other species (e.g. Rewers and Jedrzejczyk 2016; Ducar et al. 2018; Jedrzejczyk and Rewers 2018; Jedrzejczyk 2020). So far, only chromosome number and morphology have been used to support the taxonomic classification of *Trigonella* genus (Yilmaz et al. 2009; Martin et al. 2011a, 2011b; Najafi et al. 2013; Ranjbar and Hajmoradi 2016). Most species of this genus are diploids with the number of chromosomes 2n = 2x = 14 or 2n = 2x = 16 (Martin et al. 2011a, 2011b) though polyploid species/cytotypes were also observed (Malhotra 2011).

Among molecular markers, various DNA fingerprinting techniques have been used to identify species/accessions as well as to study the genetic diversity and relationships between and within different plant species/cytotypes (Rewers and Jedrzejczyk 2016; Ducar et al. 2018; Jedrzejczyk and Rewers 2018; Jedrzejczyk 2020; Jedrzejczyk and Rewers 2020). In the *Trigonella* genus, most of the molecular research were focused on the application of molecular markers like RAPD, AFLP, ITS-rDNA, SRAP, ISSR, SSR and SCoT to assess the genetic diversity and population structure of *T. foenum-graecum* (e.g. Dangi et al. 2004, 2016; Kumar et al. 2012; Randhawa et al. 2012; Tomar et al. 2014; Hora et al. 2016; Amiriyan et al. 2019; Maloo et al. 2020). So far, only Dangi et al. (2016) have provided evidence of phylogenetic relationships between 22 *Trigonella* species using nuclear ribosomal ITS and chloroplast trnL intron sequences. The research provided strong support for the monophyly of the genus and revised the previous classification of the genus.

Among different molecular marker systems, the Start Codon Targeted (SCoT) polymorphism method was proved to be suitable for plant identification and assessing their genetic diversity and relationships between genotypes. SCoT markers are based on the amplification of short, conserved regions in plant genes surrounding the translation start (or initiation) ATG codon (Collard and Mackill 2009). The markers have been successfully used to evaluate genetic diversity and structure in for instance: wheat, sugarcane and coneflower (Que et al. 2014; Etminan et al. 2016; Jedrzejczyk 2020). Daneshmand et al. (2017) investigated the genetic diversity within and between different populations of *Trigonella foenum-graecum* using ISSR and SCoT markers as well as phytochemical profiles, detecting a high level of genetic variation among them. Moreover, it was proved that the SCoT technique is more informative, polymorphic and repetitive than ISSR or RAPD markers for the evaluation of genetic diversity and relationships among fenugreek populations different in respect of trigonelline content characteristic phytochemical for seeds and leaves of fenugreek (Daneshmand et al. 2017).

The study aimed to develop a reliable marker system to support the morphological and phytochemical analysis in *Trigonella* taxonomic research, to identify and characterize species as well as determine the interspecific variation within this genus along with relationships between accessions. For this purpose, we applied analysis of genome size, endopolyploidy level in seeds, and SCoT molecular markers. Additionally, to our knowledge, this is the first report on combining the genome size estimation, endopolyploidy analysis and SCoT markers for exploring a great number of *Trigonella* species.

## Materials and methods

### Plant material

Seeds of twenty *Trigonella* accessions were received from GRIN-ARS-USDA gene bank (Table 1). To obtain plant material, all seeds were sown in 12 cm pots, mixed with sand and commercial humus (1:2, w/w), and placed in a growth chamber at 26/18°C (day/night) with 16/8 photoperiod.

**Table 1:** Genome size of the studied *Trigonella* accessions

Taxon	Section	Accession no.	Origin	DNA content (2C)			Mean CV sample %
				pg ± SD^*^		Mbp	
*T. anguina*	*Falcatulae*	PI 226534	Iran	4.02±0.07	b^*^	3932	4.55
*T. arabica*	*Pectinatae*	PI 321414	Israel	1.10±0.01	k	1076	4.61
*T. balansae*	*Falcatulae*	PI 222211	Afghanistan	1.79±0.03	i	1751	3.74
*T. calliceras*	*Callicerates*	PI 340801	Canada	2.22±0.02	f	2171	4.21
*T. caerulea*	*Capitatae*	PI 186283	Australia	1.93±0.01	h	1887	4.19
*T. coerulescens*	*Foenum-graecum*	PI 314398	Russia	2.03±0.02	g	1985	4.62
*T. corniculata*	*Falcatulae*	PI 216049	India	1.77±0.02	i	1731	3.57
*T. cretica*	*Samaroideae*	PI 340802	Canada	1.74±0.11	i	1702	4.75
*T. foenum-graecum*	*Foenum-graecum*	PI 170834	Turkey	5.76±0.02	a	5633	4.41
*T. glabra*	*Falcatulae*	PI 340803	UK	1.74±0.01	i	1702	3.55
*T. glabra* subsp. *uncata*	*Falcatulae*	PI 226533	Iran	1.80±0.01	i	1760	3.94
*T. gracilis*	*Ellipticae*	PI 540440	Pakistan	2.61±0.01	de	2553	4.00
*T. kotschyi*	*Cylindricae*	PI 206775	Turkey	4.00±0.04	b	3912	3.69
*T. macrorrhyncha*	*Foenum-graecum*	PI 222232	Iran	2.00±0.03	gh	1956	4.83
*T. schlumbergeri*	*Erosae*	PI 464828	Turkey	2.59±0.01	e	2533	3.01
*T. spicata*	*Uncinatae*	PI 206284	Turkey	2.03±0.02	g	1985	3.70
*T. spruneriana*	*Cylindricae*	PI 660996	Turkmenistan	2.17±0.03	f	2122	3.56
*T. spruneriana* ssp. *sibthorpii*	*Cylindricae*	PI 352710	Turkey	2.80±0.25	c	2738	4.27
*T. stellata*	*Falcatulae*	PI 227048	Iran	1.34±0.03	j	1310	4.67
*T. suavissima*	*Falcatulae*	PI 198170	Australia	2.67±0.01	d	2611	3.90

^*^a–k, values (in columns) followed by the same letter are not significantly different at *P* < 0.05 (Duncan’s test)

### Genome size measurements

Nuclear DNA content was estimated in fresh and young leaves of all *Trigonella* accessions (Table 1). Plant material for the flow cytometric analysis was prepared according to the protocol described by Rewers and Jedrzejczyk (2016). Two internal standards were applied for genome size calculation. For three species (*T. anguina, T. kotschyi* and *T. spruneriana* ssp*. sibthorpii*) leaves of *T. corniculata* (1.77 pg/2C) were used as the internal standard, while for the 17 species leaves of *Vicia villosa* ‘Minikowska’ (3.32 pg/2C, Dzialuk et al. 2007) were applied. The nuclei suspension was prepared using 1 ml of nuclei isolation buffer (2.5 mM MgCl_2_×6H_2_O, 85 mM NaCl, 0.1 M Tris, 0.1% (v/v) Triton X-100, pH 7.0; Rewicz et al. 2018), with the addition of propidium iodide (PI, 50 μg/ml), ribonuclease A (RNase A, 50 μg/ml) and 1.0% (w/v) polyvinylpyrrolidone (PVP-10). Samples were analysed using a CyFlow SL Green (Partec GmbH, Münster, Germany) flow cytometer, equipped with a high-grade solid-state laser, with green light emission at 532 nm, as well as with side (SSC) and forward (FSC) scatters. The DNA content of 5000-7000 nuclei was measured for each accession using linear amplification. The histograms (Mean CV = 3.01-4.83%; Table 1) were evaluated using FloMax program (Partec GmbH, Münster, Germany). Analyses were performed on six individuals per species. Genome size was determined using the linear relationship between the ratio of the 2C peak positions of *Trigonella* accessions and the internal standard on the histogram of fluorescence intensities. The 2C DNA contents (pg) were converted to the megabase pairs (Mbp) of nucleotides, using the formula: 1 pg = 978 Mbp (Doležel and Bartoš 2005; Table 1). The results have been estimated using a one-way variance analysis and Duncan’s test (*P* < 0.05; Statistica v. 13.3, StatSoft, Poland).

### Cell cycle analysis

The cell cycle was analyzed in mature, dry seeds of all accessions using the flow cytometric method (Table 2). Samples for the analysis were prepared as previously described by Rewers et al. (2009), using nuclei isolation buffer (2.5 mM MgCl_2_×6H_2_O, 85 mM NaCl, 0.1 M Tris, 0.1% (v/v) Triton X-100, pH 7.0) supplemented with 4’,6-diamidino-2-phenylindole (DAPI; 2 μg/mL) for DNA staining. Analyses were performed on five biological replicates using a CyFlow Ploidy Analyser flow cytometer (Sysmex-Partec GmbH, Gorlitz, Germany). For each sample fluorescence of 5000–7000 nuclei was recorded. The proportion of nuclei with different DNA contents, number of endocycles, and mean C-value (Lemontey et al. 2000) were calculated. In this work, only nuclei with DNA content higher than 4C were considered endopolyploid (Rewers and Sliwinska 2012, 2014; Ducar et al. 2018). The results were statistically analyzed using a one-way variance analysis and Duncan’s test (*P* < 0.05; Statistica v. 13.3, StatSoft, Poland).

**Table 2:** Percentage of nuclei with different DNA content, number of endocycles and mean C-value in seeds of the *Trigonella* species

Taxon	Percentage of nuclei with particular DNA content			Number of endocycles	Mean C-value	
	2C	4C	8C			
*T. anguina*	78	20	2	1	2.50 ± 0.28	fgh*
*T. arabica*	33	56	11	1	2.92 ± 0.22	bc
*T. balansae*	50	48	2	1	3.10 ± 0.14	b
*T. calliceras*	78	22		0	2.44 ± 0.09	gh
*T. caerulea*	67	33		0	2.67 ± 0.07	def
*T. coerulescens*	56	42	1	1	2.92 ± 0.11	bc
*T. corniculata*	71	28	2	1	2.65 ± 0.09	defg
*T. cretica*	37	61	2	1	3.32 ± 0.23	a
*T. foenum-graecum*	71	28	1	1	2.63 ± 0.23	defg
*T. glabra*	74	24	1	1	2.57 ± 0.08	efg
*T. glabra* subsp. *uncata*	51	45	3	1	3.09 ± 0.13	b
*T. gracilis*	77	23		0	2.47 ± 0.06	fgh
*T. kotschyi*	83	17		0	2.34 ± 0.27	h
*T. macrorrhyncha*	42	54	4	1	3.31 ± 0.11	a
*T. schlumbergeri*	70	27	3	1	2.72 ± 0.08	cde
*T. spicata*	72	28		0	2.57 ± 0.14	efg
*T. spruneriana*	65	32	3	1	2.82 ± 0.17	cd
*T. spruneriana* ssp. *sibthorpii*	62	38		0	2.76 ± 0.10	cde
*T. stellata*	53	43	4	1	3.09 ± 0.18	b
*T. suavissima*	53	44	3	1	3.07 ± 0.11	b

^*^a–h, values (in columns) followed by the same letter are not significantly different at *P* < 0.05 (Duncan’s test)

### DNA isolation and quantification

Total genomic DNA was extracted from 0.12 g of fresh leaf tissue of three randomly selected plants per accession, using a GeneJET Plant Genomic DNA Purification Mini Kit (Thermo Fischer Scientific, USA). DNA quality and quantity were estimated using a BioPhotometer (Eppendorf, Poland) and agarose gel electrophoresis on 1% agarose gel. Samples with high-quality DNA were used for SCoT-PCR reactions.

### DNA amplification

SCoT-PCR analyses were performed using 20 primers (Genomed, Poland), out of which 13 generated stable band patterns and were selected for further studies (Table 3). Amplification was performed in a total reaction mixture of 12 μl, containing 30 ng of genomic DNA template, 5 μl of 2x PCR Master Mix Plus (containing 0.1 U/μl Taq DNA polymerase, 4 mM MgCl_2_ and 0.5 mM of each dNTPs; A&A Biotechnology, Poland), 1 μl of 10 μM primer and sterile, deionized water. The PCR reactions were run using T100 Thermal Cycler (Bio-Rad, USA) at 94°C for 5 min, followed by 35 cycles of denaturation at 94°C for 1 min, primer annealing at 49.0-63.4°C (depending on the primer) for 1 min., primer elongation at 72°C for 2 min. The final extension step was 7 min at 72°C. The PCR products were detected on 1.5% (w/v) agarose gel stained with ethidium bromide (0.5 μg/ml). A DNA ladder of 3000 bp was used to determine the size of the amplicons (Genoplast Biochemicals, Poland). The bands were visualized and archived using GelDoc XR+ (Bio-Rad, USA).

**Table 3:** Characteristics of SCoT primers used in molecular analysis of *Trigonella* accessions

Primer code	Primer sequence (5’-3’)	Annealing temperature (°C)	No. of total alleles	No. of polymorphic alleles	Percentage of polymorphism	PIC
SCoT-2	CAACAATGGCTACAACCC	51.0	22	22	100	0.36
SCoT-5	CAACAATGGCTACAACGA	50.0	35	35	100	0.35
SCoT-6	CAACAATGGCTACCACGC	51.0	28	28	100	0.28
SCoT-7	CAACAATGGCTACCACGG	51.0	33	33	100	0.28
SCoT-9	CAACAATGGCTACCACGT	50.0	29	29	100	0.26
SCoT-11	AAGCAATGGCTACCACCA	50.0	28	28	100	0.36
SCoT-12	ACGACATGGCGACCAACG	56.0	28	28	100	0.36
SCoT-14	AGGACATGGCGACCACGC	56.0	28	28	100	0.34
SCoT-26	ACCATGGCTACCACCGTC	54.0	30	30	100	0.30
SCoT-27	ACCATGGCTACCACCGTG	54.0	31	31	100	0.34
SCoT-34	ACCATGGCTACCACCGCA	54.0	33	33	100	0.42
SCoT-35	CATGGCTACCACCCGCCC	63.5	33	32	97	0.36
SCoT-36	GCAACAATGGCTACCACC	51.0	22	22	100	0.33
Mean			**29**	**29**	**99.8**	**0.33**

### Data analysis

The banding pattern of the SCoT markers was scored as presence (1) and absence (0) of the band and set in a binary matrix. Only clear and unambiguous fragments for each primer were recorded. The numbers of monomorphic and polymorphic DNA fragments amplified by each primer were determined. The informativeness of the primer was described using the Polymorphism Information Content (PIC), and calculated according to Ghislain et al. (1999) by the formula: PIC = 1 – *p*^2^ – *q*^2^, where *p* is the band frequency, and *q* is no band frequency. According to Nei and Li (1979), genetic distances were calculated for all accessions. The phylogenetic tree was constructed using the unweighted pair group method, with arithmetic average (UPGMA), using the Treecon ver. 3.1 software (Van de Peer and De Wachter 1994). Bootstrapping was performed using 2000 replicates to assess the confidence values of the clusters formed.

## Results

### 2C DNA content

**Fig. 1:**
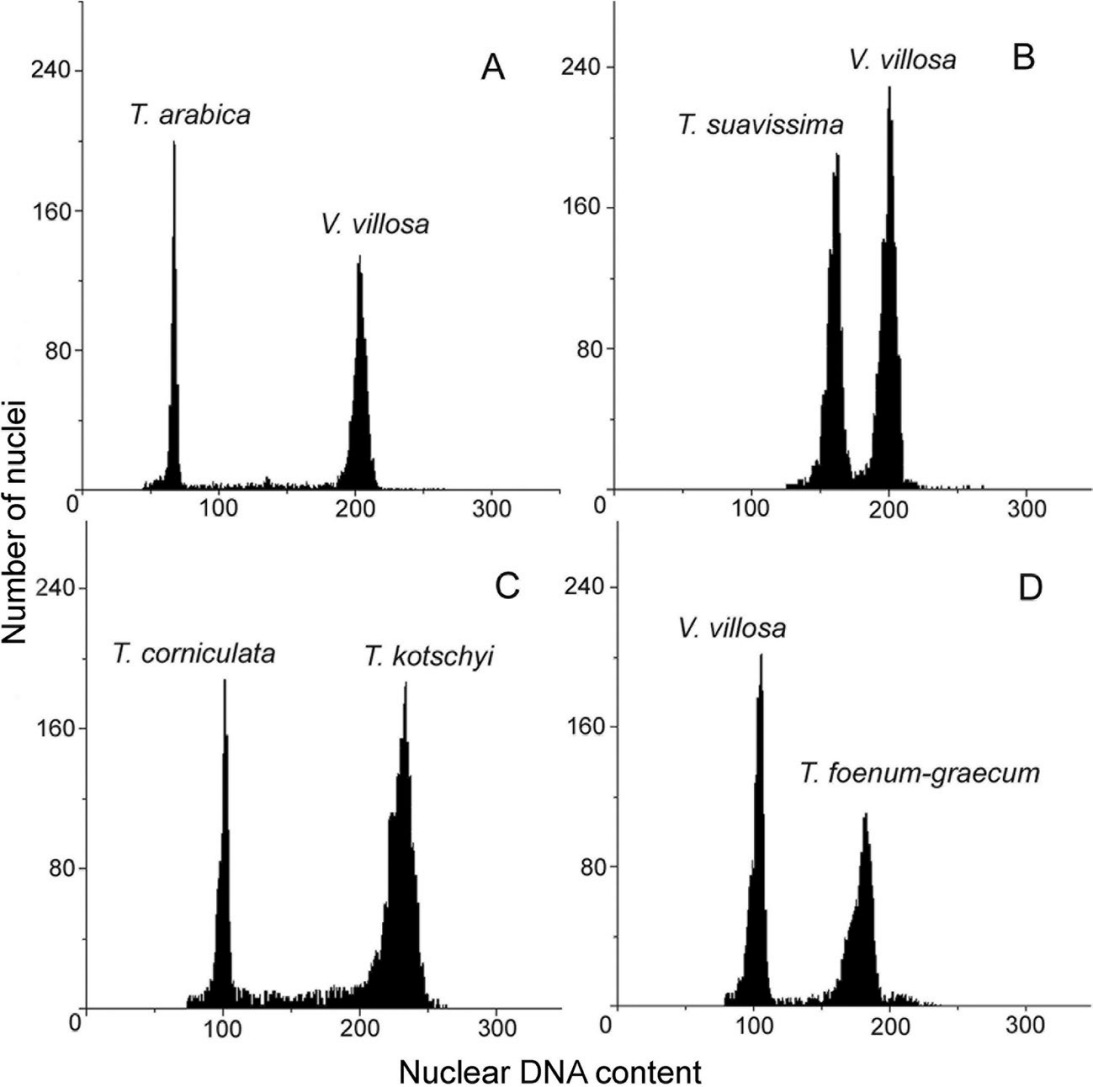
Selected histograms of the nuclear DNA content of *T. arabica* (**A**), *T. suavissima* (**B**), *T. kotschyi* (**C**) and *T. foenum-graecum* (**D**). Species *T. corniculata* and *V. villosa* were used as an internal standards

Flow cytometric analysis revealed that the genome size of *Trigonella* accessions ranged from 1.10 pg/2C in *T. arabica* (section *Pectinatae*) to 5.76 pg/2C in *T. foenum-graecum* (section *Foenum-graecum*)*,* so the difference between the smallest and the largest nuclear DNA content was 5-fold (Table 1, Fig. 1). This corresponds to 1076 and 5633 Mbp, respectively. Based on the 2C DNA content, four out of 20 studied species could be distinguished.

### Cell cycle

Based on the flow cytometric analysis, in mature and dry seeds of the studied species, endopolyploid nuclei were detected in 14 out of the 20 species examined. Only six species (*T. calliceras*, *T. caerulea*, *T. gracilis*, *T.kotschyi*, *T. spicata*, *T. spruneriana* ssp. *sibthorpii*) possess non-polysomatic seeds where only nuclei with 2C and 4C DNA content were observed. In polysomatic *Trigonella* seeds, besides nuclei with 2C and 4C, also nuclei with 8C DNA content were detected, indicating the occurrence of one endocycle. However, these species varied in the proportion of 2C, 4C and 8C nuclei. The percentage of 8C nuclei ranged from 1% (*T. coerulescens*, *T. foenum-graecum*, *T. glabra*) up to 11% (*T. arabica*). The mean C-value varied from 2.3 (*T. kotschyi*) to over 3.3 (*T. creatica*, *T. macrorrhyncha*; Table 2), revealing the lowest and the highest intensity of DNA synthesis in seeds.

### SCoT markers

**Fig. 2:**
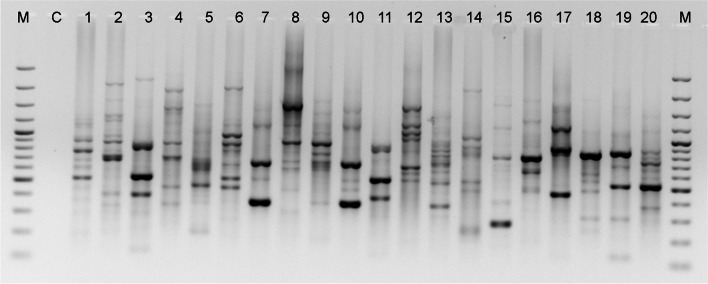
Selected SCoT band profiles of *Trigonella* accessions generated by primer SCoT-14. M. 3000 bp ladder; C. negative control; 1 -*T. anguina*, 2 - *T. arabica*, 3 - *T. balansae*, 4 - *T. calliceras*, 5 - *T. caerulea*, 6 - *T. coerulescens*, 7 - *T. corniculata*, 8 - *T. cretica*, 9 - *T. foenum-graecum*, 10 - *T. glabra*, 11 - *T. glabra* subsp. *uncata*, 12 - *T. gracilis*, 13 - *T. kotschyi*, 14 - *T. macrorrhyncha*, 15 - T*. schlumbergeri,* 16 - *T. spicata*, 17 - *T. spruneriana*, 18 - *T. spruneriana* ssp. *sibthorpii*, 19 - *T. stellata*, 20 - *T. suavissima*

A collection of 20 SCoT primers was employed to screen *Trigonella* species, and 13 of these primers generated consistent polymorphic banding patterns. The chosen primers generated 380 bands. The 12 primers revealed 100% polymorphism. The mean percentage of polymorphism for tested SCoT primers exceeded 99.8% (Table 3). The approximate size of the amplified products ranged from 174 (SCoT-35) to 2924 bp (SCoT-14). The lowest number of bands (22) were generated by SCoT-2 and SCoT-36 primers, while the SCoT-5 primer generated the highest number of bands (35), all polymorphic. The PIC values varied between 0.26 (SCoT-9) to 0.42 (SCoT-34), with an average of 0.33 (Table 3). Four primers: SCoT-7, SCoT-11, SCoT-12, and SCoT-14 were the most effective in species distinction, whereas in genotypes diversification the most accurate were primers: SCoT-7, SCoT-11 and SCoT-14 (the distinction between *T. glabra* and *T. glabra* subsp. *uncata;* Fig. 2), and SCoT-11, SCoT-12 and SCoT-14 (between *T. spruneriana* and *T. spruneriana* ssp. *sibthorpii*).

**Fig. 3:**
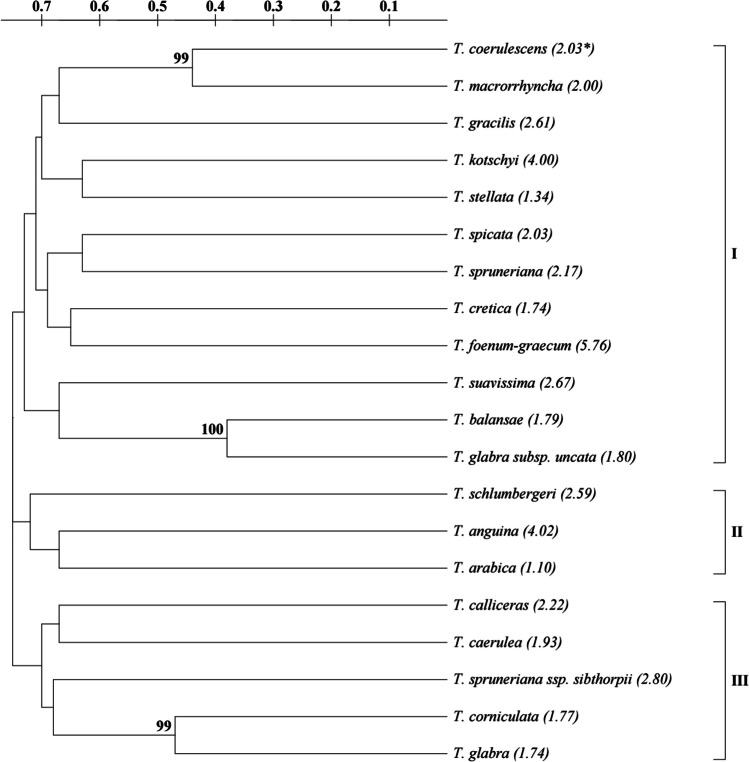
The phylogenetic relationships of the studied fenugreek species based on UPGMA analysis of SCoT markers. Only bootstrap values >50% are indicated; scale indicates genetic distance. *genome size (pg/2C) are indicated in the brackets

The genetic distance analysis between studied accessions revealed that the highest genetic similarity (0.38) was observed between *T. balansae* and *T. glabra* subsp*. uncata*, whereas the lowest similarity (0.84) was found between *T. corniculata* and *T. spicata,* as well as *T. arabica* and *T. caerulea* (Table 4). The dendrogram was constructed using binary matrix values as determined from SCoT data and grouped all studied species into three main clusters. The first cluster (I) included 12 species, while the second one (II) had only three species, and the third cluster (III) comprised five species (Fig. 3).

**Table 4:** Genetic distance matrix based on SCoT markers among 20 *Trigonella* accessions

No.	1	2	3	4	5	6	7	8	9	10	11	12	13	14	15	16	17	18	19	20
1	0.00																			
2	0.67	0.00																		
3	0.82	0.77	0.00																	
4	0.74	0.76	0.74	0.00																
5	0.77	0.84	0.72	0.67	0.00															
6	0.69	0.74	0.74	0.78	0.75	0.00														
7	0.69	0.76	0.72	0.71	0.71	0.74	0.00													
8	0.74	0.83	0.71	0.75	0.72	0.72	0.76	0.00												
9	0.67	0.71	0.69	0.75	0.76	0.72	0.74	0.65	0.00											
10	0.74	0.80	0.73	0.66	0.72	0.78	0.47	0.67	0.75	0.00										
11	0.79	0.76	0.38	0.72	0.73	0.70	0.74	0.75	0.70	0.71	0.00									
12	0.71	0.74	0.74	0.80	0.74	0.68	0.75	0.70	0.66	0.70	0.68	0.00								
13	0.69	0.74	0.80	0.76	0.68	0.65	0.77	0.68	0.76	0.78	0.76	0.70	0.00							
14	0.70	0.74	0.79	0.81	0.76	0.44	0.81	0.69	0.78	0.75	0.77	0.66	0.68	0.00						
15	0.69	0.76	0.82	0.77	0.81	0.80	0.79	0.81	0.77	0.82	0.78	0.80	0.74	0.72	0.00					
16	0.74	0.68	0.79	0.81	0.74	0.69	0.84	0.71	0.73	0.83	0.78	0.76	0.68	0.71	0.68	0.00				
17	0.71	0.81	0.75	0.79	0.71	0.69	0.79	0.68	0.63	0.75	0.72	0.66	0.65	0.68	0.80	0.63	0.00			
18	0.75	0.79	0.80	0.73	0.69	0.72	0.66	0.70	0.80	0.70	0.75	0.80	0.64	0.68	0.72	0.70	0.79	0.00		
19	0.76	0.72	0.70	0.78	0.70	0.72	0.72	0.74	0.70	0.71	0.64	0.68	0.63	0.74	0.79	0.74	0.72	0.72	0.00	
20	0.68	0.79	0.69	0.77	0.77	0.75	0.73	0.71	0.71	0.75	0.65	0.69	0.68	0.75	0.71	0.75	0.64	0.78	0.72	0.00

1 -*T. anguina,* 2 - *T. arabica,* 3 - *T. balansae,* 4 - *T. calliceras,* 5 - *T. caerulea,* 6 - *T. coerulescens,* 7 - *T. corniculata,* 8 - *T. cretica,*9 - *T. foenum-graecum,* 10 - *T. glabra,* 11 - *T. glabra* subsp. *uncata,* 12 - *T. gracilis,* 13 - *T. kotschyi,* 14 - *T. macrorrhyncha,* 15 - *T. schlumbergeri,* 16 - *T. spicata,* 17 - *T. spruneriana,* 18 - *T. spruneriana* ssp*. sibthorpii,* 19 - *T. stellata,* 20 - *T. suavissima*

## Discussion

The *Trigonella* genus contains species that are important as medicinal and aromatic plants and are used for multiple purposes. Fresh, dry or powdered leaves, seeds, and extracts induce various pharmacological effects. Therefore, the knowledge about genome size, genetic diversity and the relationship between the species could be very useful in the proper utilization of germplasm resources as well as for its usage in the pharmaceutical industry and breeding programs of *Trigonella* species.

In this study, the species from 10 sections of *Trigonella* genus were investigated. The most numerous section *Falcatulae,* was represented by seven species, while the sections *Cylindricae* and *Foenum-graecum* by three species. In the other seven sections (*Callicerates, Capitatae, Ellipticae, Erosae, Pectinatae, Samaroideae, Uncinatae*), only one species was represented. The genome size measured for seven species of *Falcatulae* section ranged from 1.34 to 4.02 pg/2C. For three species from *Cylindricae* section, the 2C DNA content varied from 2.17 to 4.00 pg/2C, while among species of *Foenum-graecum* section, the values of DNA content ranged from 2.00 to 5.76 pg/2C. In the sections *Cellicerates, Ellipticae, Erosae* and *Uncinatae*, the genome size ranged from 2.03 to 2.61 pg/2C, whereas in *Pectinatae, Capitatae* and *Samaroideae* was below 2.00 pg/2C. The genome size estimated for 20 *Trigonella* species could be used to screen genetic diversity within the genus and as an additional parameter for species identification. The nuclear DNA content estimated by FCM analysis allowed to identify four species (*T. arabica*, *T. foenum-graecum*, *T. spruneriana* ssp. *sibthorpii* and *T. stellata*). To the best of our knowledge, the 2C DNA content for 19 species of *Trigonella* genus was estimated for the first time. So far, only for *T. stellata* the 2C DNA content was measured using the Feulgen method (Bidak and Brandham 1995) and was only 0.06 pg/2C higher than obtained by FCM in the presented study (1.34 pg/2C). According to the categorization proposed by Soltis et al. (2003), 17 species can be considered as having very small genomes (1.10-2.80 pg/2C), while three species as possessing small genome sizes (4.00-5.76 pg/2C). The usefulness of FCM method in plant identification and determination of genetic diversity has also been proved in many herbal plants, e.g. representing *Echinacea* (Jedrzejczyk 2020), *Mentha* (Jedrzejczyk and Rewers 2018), *Origanum* (Jedrzejczyk 2018) and *Malva* genera (Jedrzejczyk and Rewers 2020). Based on the literature (Martin et al. 2011a, 2011b; Ranjbar and Hajmoradi 2016), most of the studied *Trigonella* species are diploids with somatic chromosome number 2n=2x=16. Only for *T. stellata* 18 chromosomes were reported in somatic cells. There is no information about the chromosome number for two species, T*. schlumbergeri* and *T. suavissima*. Since the occurrence of extra B chromosomes in some fenugreek lines, the variation in chromosome number can also be observed (Raghuvanshi and Singh 1976; Petropoulos 2002; Martin et al. 2011a, 2011b; Ranjbar and Hajmoradi 2016).

Despite that the *Trigonella* genus belongs to the Fabaceae family, known for high polysomaty, this genus represented a low level of endopolyploidy in seeds. Among the investigated species, two groups could be distinguished based on the cell cycle analysis in seeds. The first group included 14 species with polysomatic seeds where one endocycle occurred and nuclei with DNA content up to 8C were present. While in the second group, six species with non-polysomatic seeds were included. In seeds of this group, no endocycles were observed, and only nuclei with 2C and 4C were detected. Application of this feature in species identification (together with genome size estimation) enables the distinction of the seven next species. Altogether 11 species could be identified based on flow cytometric measurements. Therefore, endopolyploidy level in seeds can be considered a supportive feature for species identification and classification. Additionally, this is the first report on endopolyploidy in *Trigonella* seeds.

The *Trigonella* genus variability analysis using different molecular methods (RAPD, ISSR, SCoT, SSR, SCAR, ITS) is mostly concentrated on *T. foenum-graecum* genotypes (Mirzahosein-Tabrizi et al. 2023). Studies on *T. foenum-graecum* concerning the application of RAPD markers recorded polymorphism between 43 and 92% (Hora et al. 2016; Mamatha et al. 2017) while for *T. caerulea* 95% (Dangi et al. 2004). In the case of ISSR markers, the percentage of polymorphism obtained for *T. foenum-graecum* populations was between 31 and 92% (Marzougui et al. 2009; Randhawa et al. 2012). Only Al-Maamari et al. (2014) reported 99-100% of polymorphism in *T. foenum-graecum* from Oman using six AFLP markers. This was higher than the values obtained by Kumar et al. (Kumar et al. 2012; 64%). Nevertheless, to our knowledge, this is the first report of using SCoT molecular markers for exploring genetic diversity for such a large group of species from *Trigonella* genus. In this study, SCoT markers revealed a high level of polymorphic loci (99.8 %) among all investigated species, being higher than results obtained for *T. foenum-graecum* accessions (82%) using the same markers (Daneshmand et al. 2017). The efficiency of SCoT markers was also estimated based on Polymorphism Information Content, and the obtained value was 0.33, which was similar to PIC estimated by Daneshmand et al. (2017). The significant level of genetic divergence has been calculated by SCoT molecular markers in other herbal plants, e.g. from *Echinacea* genus (Jedrzejczyk 2020), *Crepedium acuminatum* (Thakur et al. 2021), *Lycoris* species (Gao et al. 2014) and *Papaver somniferum* (Srivastava et al. 2020), what proved the potential of these markers for assessing genetic diversity and relationships in plants.

The results of SCoT-PCR analysis allowed for establishing the relationship between species, considered by genetic distance and dendrogram construction. This phylogenetic analysis discovered the existence of three clusters. The highest genetic distance was observed between *T. corniculata* and *T. spicata*, grouped into two separate clusters, I (*T. spicata*) and III (*T. corniculata*), as well as between *T. arabica* and *T. caerulea*, clustered in II and III group, respectively. The lowest genetic distance was observed between *T. balansae* and *T. glabra* subsp*. uncata,* species (included in the cluster I)*.* This indicated the highest similarity between these species. Based on genetic distance analysis and genome size, we could not reach a consensus on the parameters. In the I cluster, both the highest and the lowest values of genome sizes were found. However, closely related species (e.g. *T. corniculata* and *T. glabra*) possess similar genome sizes. Most of the tested primers generated polymorphic bands. However, three proved to be the best for all the species distinction. Moreover, it was also possible to indicate the primers for distinguishing the genotypes of *T. glabra* and *T. glabra* subsp. *uncata* as well as *T. spruneriana* and *T. spruneriana* ssp. *sibthorpii*. This supports previous findings of high-resolution of SCoT markers in analyzing the genetic diversity of species from *Echinacea* genus (Jedrzejczyk 2020) and the Fabaceae family (Nosair 2016).

## Conclusions

The results obtained in the study revealed that the *Trigonella* genus could be characterized using SCoT molecular markers with the support of genome size estimation and cell cycle analysis performed by FCM. The presented results increased the knowledge about *Trigonella* genus, particularly its genome size, endopolyploidy and genetic diversity, as well as phylogenetic relationships between the *Trigonella* species. This could be useful in the conservation of medicinal plants and the application of some species in breeding programs.

## Data Availability

The data that support the findings of this study are available from the corresponding author, [MR], upon reasonable request.
